# Icariin improves testicular dysfunction via enhancing proliferation and inhibiting mitochondria-dependent apoptosis pathway in high-fat diet and streptozotocin-induced diabetic rats

**DOI:** 10.1186/s12958-021-00851-9

**Published:** 2021-11-09

**Authors:** Weiguo He, Huiqing Liu, Linlin Hu, Yaohui Wang, Lane Huang, Aihong Liang, Xuan Wang, Qing Zhang, Yi Chen, Yi Cao, Suyun Li, Junli Wang, Xiaocan Lei

**Affiliations:** 1 Clinical Anatomy and Reproductive Medicine Application Institute, Hengyang Medical School, University of South China, Hengyang, 421001 China; 2 Reproductive Medicine Center, The Affiliated Hospital of Youjiang Medical University for Nationalities, Baise, 533000 China; 3 School of Basic Medical Sciences, Zunyi Medical University, Zunyi, 563000 China

**Keywords:** Icariin, Reproductive protection, Spermatogenesis, Proliferation, Apoptosis

## Abstract

**Background:**

Diabetes mellitus (DM), a chronic metabolic disease, severely impairs male reproductive function. However, the underpinning mechanisms are still incompletely defined, and there are no effective strategies or medicines for these reproductive lesions. Icariin (ICA), the main active component extracted from *Herba epimedii*, is a flavonoid traditionally used to treat testicular dysfunction. Whether ICA can improve male reproductive dysfunction caused by DM and its underlying mechanisms are still unclear. In this study, by employing metformin as a comparative group, we evaluated the protective effects of ICA on male reproductive damages caused by DM and explored the possible mechanisms.

**Methods:**

Rats were fed with a high fat diet (HFD) and then intraperitoneally injected with streptozotocin (STZ) to induce diabetes. Diabetic rats were randomly divided into T2DM + saline group, T2DM + metformin group and T2DM + ICA group. Rats without the treatment of HFD and STZ were used as control group. The morphology of testicular tissues was examined by histological staining. The mRNA expression levels were determined by quantitative real-time PCR. Immunostaining detected the protein levels of proliferating cell nuclear antigen (PCNA), hypoxia-inducible factor 1-alpha (HIF-1α) and sirtuin 1 (SIRT1) in testicular tissues. TUNEL assay was performed to determine cell apoptosis in the testicular tissues. The protein expression levels of HIF-1α and SIRT1 in the testicular tissues were determined by western blot assay.

**Results:**

ICA effectively improved male reproductive dysfunction of diabetic rats. ICA administration significantly decreased fasting blood glucose (FBG) and insulin resistance index (IRI). In addition, ICA increased testis weight, epididymis weight, sperm number, sperm motility and the cross-sectional area of seminiferous tubule. ICA recovered the number of spermatogonia, primary spermatocytes and Sertoli cells. Furthermore, ICA upregulated the expression of PCNA, activated SRIT1-HIF-1α signaling pathway, and inhibited intrinsic mitochondria dependent apoptosis pathway by upregulating the expression of *Bcl-2* and downregulating the expression of *Bax* and *caspase 3*.

**Conclusion:**

These results suggest that ICA could attenuate male reproductive dysfunction of diabetic rats possibly via increasing cell proliferation and decreasing cell apoptosis of testis. ICA potentially represents a novel therapeutic strategy against DM-induced testicular damages.

## Introduction

Diabetes mellitus (DM), characterized with hyperglycemia, is a chronic metabolic disease resulting from dysfunction in insulin secretion and/or insulin action [1, 2]. Recently, the morbidity of DM rapidly increased [3]. Owning to the complex and severe complications [3], DM has become one of the major causes of death [4], severely threatening the health and social economy of the world [5]. There is a close link between the morbidity of DM and the infertility [6]. Increasing striplings at reproductive age, even in children, have been diagnosed with DM [7]. Furthermore, growing evidence shows that hyperglycemia in DM can broadly impair male reproduction system [8–13], accompanying with severely histological damage of testis including the morphological disruption of seminiferous tubular and the disturbance of spermatogenesis [7, 11, 14], decrease in serum sexual hormone, increase in oxidative stress and apoptosis in testis [7]. However, the underlying mechanisms of male reproductive impairments caused by DM are still incompletely defined, and there are no effective strategies or medicines for these reproductive lesions [7].

Currently, screening effective drug candidates from traditional Chinese medicine has attracted increasing attention [15]. Studies have found that various compounds from herbal extracts such as alpha-lipoic acid, resveratrol and cinnamon showed protective effects on the male reproduction systems under diabetic conditions [16–18]. Icariin (ICA), the main active component of *Herba epimedii*, is a flavonoid traditionally be used to treat erectile dysfunction [19, 20]. Several studies indicated that ICA administration with moderate dose could promote spermatogenesis by elevating the levels of follicular stimulating hormone receptor (FSHR) and increasing testosterone secretion via upregulating the expression of steroidogenic acute regulatory protein (StAR) and peripheral type benzodiazepine receptor (PBR), which is indispensable element of the steroidogenic machinery and functions in a coordinated manner to transfer cholesterol into mitochondria [19, 21, 22]. Moreover, Nan et al. found that ICA could stimulate the proliferation of Sertoli cells [23], which play an important role in regulating the self-renewal and differentiation of spermatogonial stem cells [24, 25]. Correspondingly, ICA also ameliorated microcystin-LR-induced and aging-related Sertoli cell injury [26, 27], then increased the epididymal sperm count [19]. These results suggest that ICA could benefit male reproductive function by regulating the hypothalamic–pituitary–gonadal (HPG) axis [28]. Some recent studies also indicated the anti-apoptotic properties of ICA in other systems [15]. In cardiomyocytes, it is reported that ICA reduced oxidative stress and ameliorated apoptosis through alleviating mitochondrial dysfunction [29–32]. Qiao et al. showed that ICA pretreatment attenuated cisplatin-induced nephrotoxicity via reducing the levels of reactive oxygen species (ROS) and suppressing mitochondria dependent apoptotic pathway [33]. Wang et al., found that ICA enhanced neuronal viability and attenuated neuronal death [34], and Zhu et al., further confirmed this conjecture [35]. Sun et al., also verified the attenuated effect of ICA on high glucose-induced cell apoptosis and oxidative stress in human umbilical venous endothelial cells [36]. Furthermore, Zhao et al., confirmed that ICA could inhibit AGE-induced PC12 cell injury by specifically targeting Bax, then further regulate mitochondrial apoptosis process [37]. These studies suggested that the anti-apoptosis effect of ICA is tightly linked to classical mitochondria apoptosis pathway [15]. Consequently, it is rational to speculate that mitochondria apoptosis pathway may involve the protective effects of ICA on male reproductive system. Indeed, the normal development of germ cells and reproductive tissues is highly dependent on balanced regulation of mitochondrial apoptosis by BCL-2 family proteins [38], and there is a correlation between increased testis damage and higher mitochondrial apoptosis in diabetic rats [14, 39]. However, the effect of ICA on male reproductive dysfunction of diabetic rats and the underlying mechanisms are still not defined.

In this study, comparing with metformin, we evaluated the protective roles of ICA on the DM-induced testicular dysfunction via establishing a high fat diet (HFD) and streptozotocin (STZ) induced diabetic rat model. We sought to identify the effect of ICA on cell proliferation and apoptosis in the testis of diabetic rats. We speculated that ICA could effectively attenuate DM-induced male reproductive damages and may provide a potentially therapeutic strategy against male infertility caused by DM.

## Materials and methods

### Animals and reagents

Male Sprague-Dawley rats (6 weeks old, body weight 170 ± 10 g), purchased from the Animal Experimental Center of Daping hospital, Third Military Medical University, were maintained in an air-conditioned animal facility with a 12 h light/dark cycle. Rats were provided with standard food pellets and tap water ad libitum, and this study was approved by the Animal Ethics Committee of University of South China (NO.. USC2020031602). Streptozotocin (STZ) was purchased from Sigma-Aldrich (S0130, St. Louis, USA), Metformin and ICA were purchased from Sigma-Aldrich (St. Louis, USA) and Xi’an Xiaocaokeji Ltd. (Xi’an, China) respectively. Rats were fed with a high fat diet (HFD, consisting of 70% standard food pellet, 10% fat and 20% sugar) and then intraperitoneally injected with STZ (45 mg/kg) to induce diabetes. Fasting blood glucose (FBG) levels, which were tested for 3 days after the injection of STZ, over 16.7 mM were considered as type 2 diabetic rats (T2DM) [14]. Then, T2DM were randomly divided into three groups including T2DM + saline group, T2DM + metformin group and T2DM + ICA group (*n* = 6 for each group). In addition, extra 6 rats without the treatment of HFD and STZ were employed as control group [14]. After scanning the optimal dose, 100 mg/kg/d of metformin (dissolved in saline solution) and 80 mg/kg/d of ICA (dissolved in 1% sodium carboxymethyl cellulose) were orally administered to T2DM for 6 weeks in T2DM + Metformin and T2DM + ICA group, respectively [40]. At the end of treatment period, all rats were anesthetized with 30 mg/kg of pentobarbital sodium before sacrifice.

### Assessment of diabetic rats

After fasting overnight, FBG and fasting blood insulin levels (FINS) were tested with a Glucose Meter (Contournext, Parsippany, USA) and an insulin kit (Abcan, Cambridge, USA), respectively. HOMA-IR was calculated with the following formula: (FBG × FINS)/22.5 [14].

### Hormonal analysis

The serum levels of testosterone, luteinizing hormones (LH) and follicle-stimulating hormone (FSH) in the rats were analyzed using the commercial kits from Elabscience® Biotechnology Inc. (Houston, USA) according to the manufacturer’s instruction [41].

### Sperm analysis

For the sperm collection, cauda epididymis of all rat testis was collected and then transferred to a sterilized Petri dish with 2 mL normal saline. Sperm counting and motility analysis were performed according to the standard procedure [14] by a masterly operator with a Sperm Class Analyzer (MICROPTIC, Barcelona, Spain).

### Histological analysis

For histology, partial testis tissue was fixed in 4% paraformaldehyde overnight. Paraffin sections (6 μm) were sequentially cut, deparaffined and further stained with hematoxylin and eosin. The microstructure of testes, counting of spermatogonia, primary spermatocytes and Sertoli cell were photographed and performed under a light microscope (OLYMPUS BX43). For the counting, 10 randomly chosen seminiferous tubules in each slide (3 slides per rat) and totally 180 seminiferous tubules were selected, and the cross-sectional area of seminiferous tubules employed were analyzed by OPLYMPUS cell Sens Standard (Version 1.15).

### Immunohistochemical analysis

Immunohistochemistry was performed according to previous study with minor modification [14]. Partial testes were fixed in 4% formaldehyde and embedded in paraffin. Then, sections (6 μm) were sequentially permeabilized with 1% Triton X-100 in phosphate buffered saline (PBS) for 30 min at room temperature, boiled in 100 mM sodium citrate (pH 6.0) three times (6 min each) with 5 min intervals for antigen retrieval, washed in 3% hydrogen peroxide for 30 min to remove endogenous peroxidase and blocked for 1 h in 5% bovine serum albumin at room temperature. After that, slides were first incubated overnight at 4 °C with primary antibodies of proliferating cell nuclear antigen (PCNA; 1:100 dilution, sc-25,280, Santa Cruz Biotechnology, CA, USA), sirtuin 1 (SIRT1; 1:100 dilution, ab189494, Abcam, Cambridge, UK) and hypoxia-inducible factor 1-alpha (HIF-1α; 1:100 dilution, ab228649, Abcam, Cambridge, UK) in the blocking solution respectively, then further rewarmed for 45 min at 3 7 °C the next day. Following three washes with 0.1% Tween-20 in PBS, the samples were incubated with rabbit anti-goat Biotin-SP-conjugated antibody (1:100 dilution, SA00004-4, Protein Tech Group Inc., Wuhan, China) in the blocking solution for 45 min at room temperature and 45 min at 37 °C, respectively. The immunoreactive signals were detected with Peroxidase-conjugated Streptavidin (1:100 dilution, SA00001–0, Protein Tech Group, Wuhan, China). The normal goat IgG (1:200 dilution) was used as a negative control. Finally, sections were observed and photographed with an Olympus DP70 digital camera mounted on a Leica DMR microscope.

### TUNEL assay

For apoptotic analysis, TUNEL assay was carried out by following the manufacturer’s instructions (Roche Diagnostic Systems, Branchburg, NJ, USA) with minor modification [14]. Briefly, sections were deparaffined and rehydrated, and then sequentially treated with proteinase K and 3% hydrogen peroxide, following the incubation with the TUNEL reaction mixture in a humid chamber at 37 °C. Then, 3, 3-diaminobenzidine chromogen were applied for the detection of peroxidase-conjugated anti-biotin antibody. Finally, slices were counterstained with Hematoxylin. Sections without TdT-enzyme treatment were used as a negative control. Quantitative analysis of apoptotic cells was performed according to previous study [14] with minor modification. Only spermatogonia, primary spermatocytes and Sertoli cells were taken into account owning to the difficulty of quantification.

### Quantitative real-time PCR (qRT-PCR) assay

Total RNA was extracted from the testes of rats with TRIzol reagent (Themo Fisher, Inc., USA). cDNA was synthesized with the TransScript II One-Step gDNA Removal and cDNA Synthesis SuperMix kit (Transgen Biotech, Beijing, China) according to the manufacturer’s protocol. By using GAPDH as the internal reference, quantitative PCR analyses were carried out in the SYBR Green assay system with the Applied Biosystems 7500 Real-time PCR System (Applied Biosystems, Foster City, CA, USA). The mRNA expression levels were calculated by using the 2^-ΔΔCt^ method. The primers used in this study were listed in Table 1.

**Table 1:** Primer pairs and corresponding annealing temperatures used in this study

Gene	Sequence(5′-3′)	Annealing Temperature	Product (bp)
PCNA	F: GCTCCATCCTGAAGAAGGT R: TGCACTAAGGAGACGTGAGA	55 °C	121
Bax	F:GAGACACCTGAGCTGACCTT	55 °C	104
	R:TCCATGTTGTTGTCCAGTTC		
Bcl-2	F:AGTACCTGAACCGGCATCT	55 °C	120
	R:TCTTCAGAGACAGCCAGGA		
Caspase-3	F:CCGGTTACTATTCCTGGAGA	55 °C	117
	R:TAACACGAGTGAGGATGTGC		
SIRT1	F:GTGGCAGTAACAGTGACAGTG R:GTCAGCTCCAGATCCTCCAG	55 °C	143
HIF1α	F:GATGGAATGGAGCAGAAGAC R: CACAATCGTAACTGGTCAGC	55 °C	112
CAT	F: ACAACTCCCAGAAGCCTAAGAATG R: RGCTTTTCCCTTGGCAGCTATG	55 °C	76
Gpx	F: GGAGAATGGCAAGAATGAAGA R: RCCGCAGGAAGGTAAAGAG	55 °C	139
SOD	F: CGAGCATGGGTTCCATGTC R: CTGGACCGCCATGTTTCTTAG	55 °C	101
GAPDH	F: CCTCAAGATTGTCAGCAATG R: CAGTCTTCTGAGTGGCAGTG	55 °C	134

### Western blotting assay

The testes of rats were lysed in RIPA buffer with 0.1% cocktail and 10% phosphotransferase inhibitor. Total protein from each sample was subjected to sodium dodecyl sulphate–polyacrylamide gel electrophoresis and subsequently transferred to nitrocellulose membranes (BIO-RAD, Hercules, CA, USA, 0.22 μM). Transferred membranes were blocked with 5% non-fat milk containing TBST (TBS containing 0.1% Tween-20) for 45 min at room temperature. Thereafter, primary antibodies including SIRT1 (1:1000 dilution, ab189494, Abcam, Cambridge, UK), HIF-1α (1:1000 dilution, ab228649, Abcam, Cambridge, UK) and β-actin (1:1000 dilution, ab8227, Abcam, Cambridge, UK) were respectively incubated overnight at 4 °C and three washing were performed before the incubation of horseradish peroxidase-conjugated goat anti-rabbit IgG (secondary antibody, 1:1500 dilution, sc-2004; Santa Cruz Biotechnology, CA, USA) for 30 min at room temperature. Finally, Bio-Rad ChemiDoc system was used to analyze the intensities of autoradiographic bands.

### Statistical analysis

Statistical analyses were performed with SPSS 17.0 (IBM, Armonk, NY, USA). The normality of data was examined by Kolmogorov-Smirnov test. One-way ANOVA test followed by Dunnett’s post hoc test were used to analyze the statistic differences among different groups. The data are presented as mean ± standard deviation (SD), and *P* < 0.05 was considered statistically significant.

## Results

### ICA ameliorated diabetic parameters of diabetic rats

**Fig. 1:**
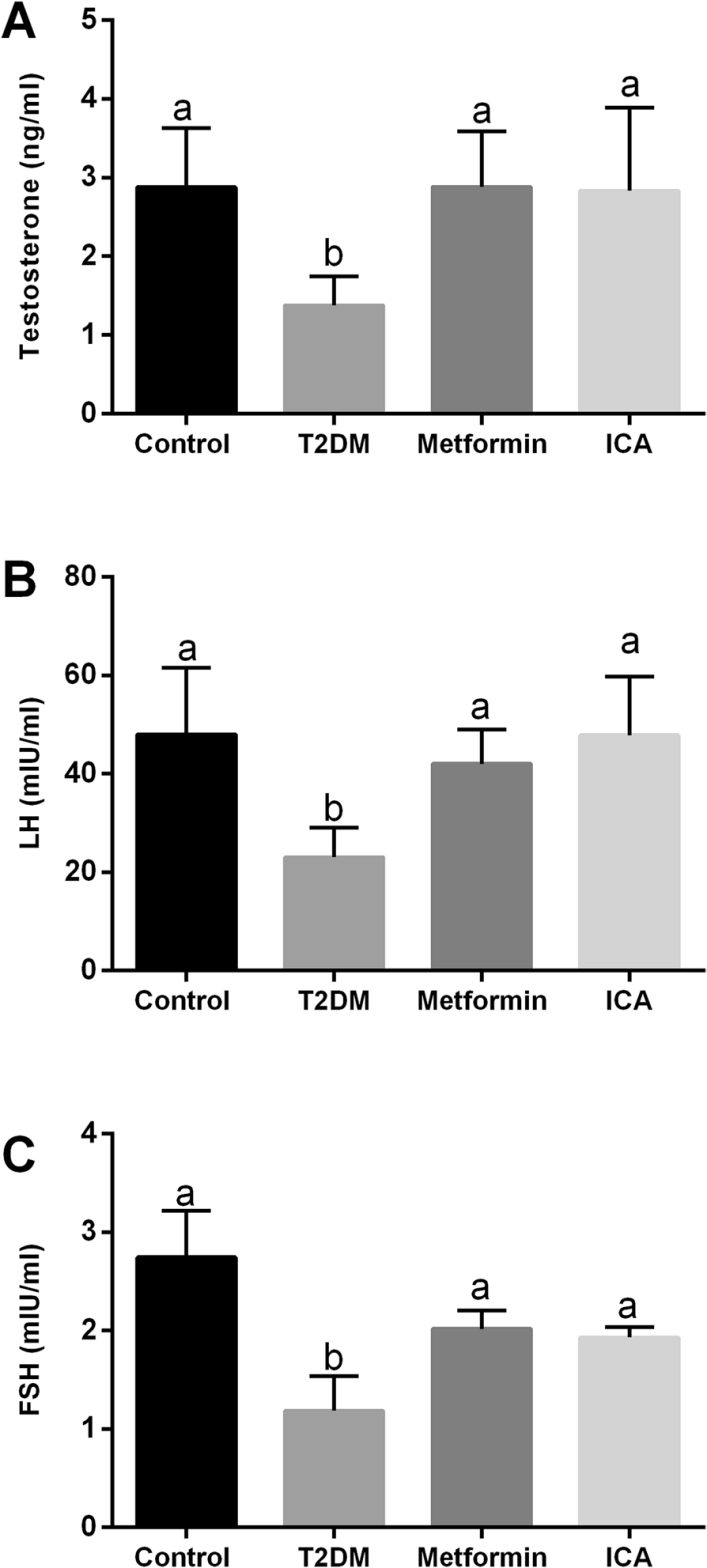
Effects of ICA administration on the serum testosterone, LH and FSH levels in the diabetic rats. **A** Testosterone, (**B**) LH and (**C**) FSH levels are shown. *N* = 6. Bars assigned with different superscripts mean significant difference (*p* < 0.05)

According to the diagnostic criteria of DM [1, 2], we successfully established T2DM rat model. These diabetic rats showed significant decrease in body weight and increase in FBG, FINS and insulin resistance index (IRI) (Table 2). To test the efficiency of ICA, we examined the same parameters abovementioned in ICA-treated diabetic rats. ICA administration observably decreased FBG and IRI, but had no effect on body weight and FINS (Table 2). As a first-line drug for the management of hyperglycaemia [42–44], metformin was also employed as a comparative group to evaluate the efficiency of ICA. Similar to previous studies [14], metformin administration increased body weight and FINS, and decreased FBG and IRI (Table 2). The hormonal analysis results showed that the serum levels of testosterone, LH and FSH were significantly lower in the T2DM group than that in the control group (Fig. 1). Metformin and ICA administration both restored the sexual hormone levels when compared to T2DM group (Fig. 1).

**Table 2:** Effect of ICA on body weight, blood glucose, insulin levels and insulin resistance index in diabetic rats

Group	BW (g)		FBG (mmol L^−1^)		FINS (uIU mL^−1^)	IRI
	Before	After	Before	After		
Control	341.2 ± 7.644^**◇**^	402.5 ± 8.705^a**◈**^	6.150 ± 0.5296^a**◇**^	4.817 ± 0.1833^a**◇**^	73.89 ± 15.00^a^	20.96 ± 0.940^a^
T2DM	316.7 ± 17.09^**◇**^	232.8 ± 26.64^b**◈**^	22.07 ± 0.7843^b**◇**^	27.48 ± 0.9630^b**◇**^	96.26 ± 40.07^b^	188.5 ± 51.80^b^
Metformin	321.2 ± 5.350^**◇**^	350.0 ± 31.54^c**◇**^	18.10 ± 0.5125^b**◇**^	5.000 ± 0.4359^a**◈**^	188.9 ± 82.10^c^	39.72 ± 19.31^c^
ICA	340.7 ± 13.20^**◇**^	261.6 ± 11.43^b**◈**^	22.43 ± 0.7796^b**◇**^	7.650 ± 2.0840^a**◈**^	118.2 ± 13.62^b^	23.24 ± 9.770^a^

In each column, values assigned with different superscript lowercases (letters) indicate significant difference (*P* < 0.05). In each row of BW and FBG, values assigned with different superscript symbols (diamond symbols) also indicate significant difference (*P* < 0.05). *BW* body weight; *FBG* fasting blood glucose; *FINS* fasting insulin; *IRI* insulin resistance index

### ICA mitigated testicular dysfunction of diabetic rats

**Fig. 2:**
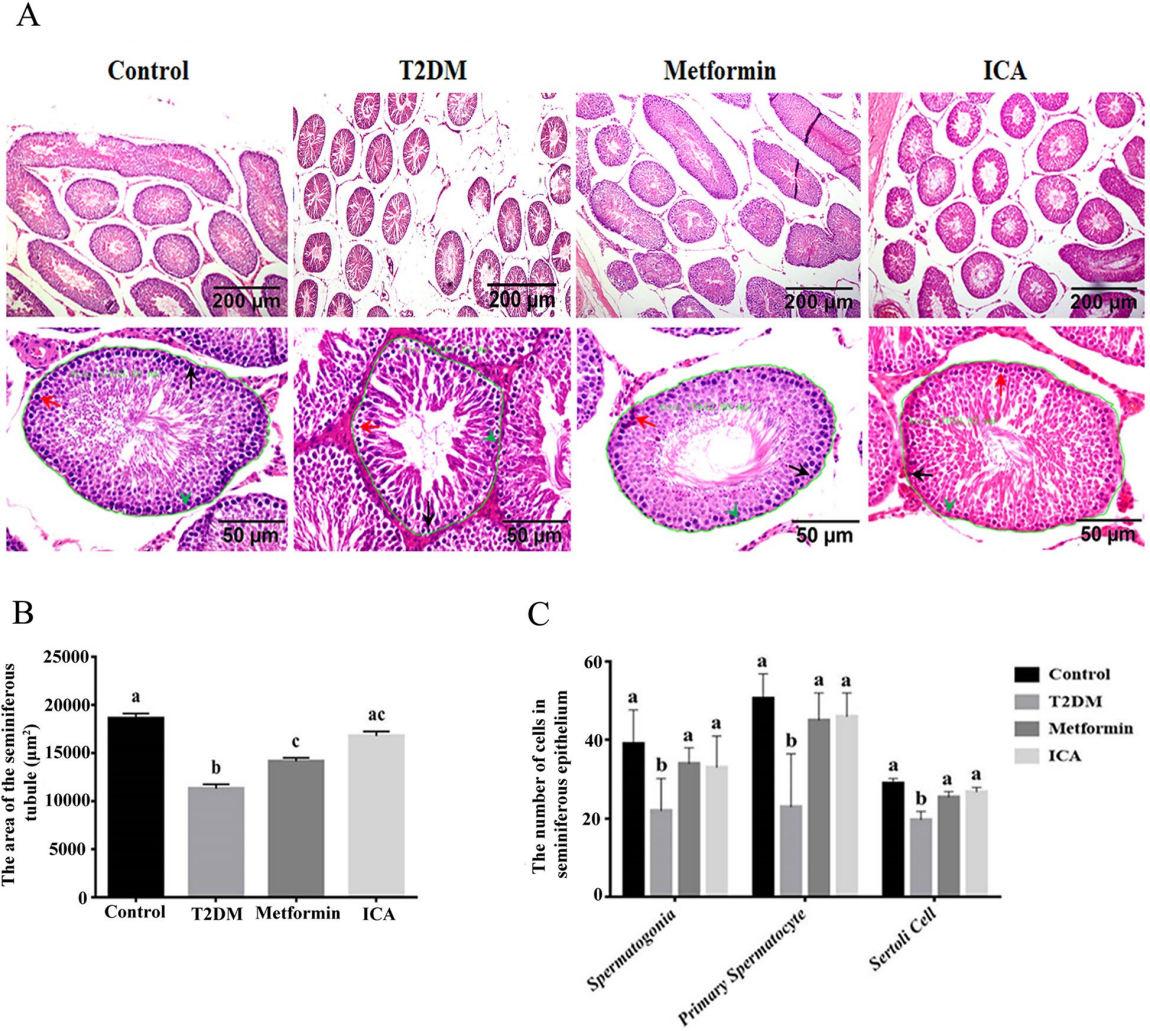
ICA administration improved impaired seminiferous tubule in diabetic rats. **A** Hematoxylin and eosin staining of testicular tissue. The lower panes show partial amplification of upper panes. In each seminiferous tubule, the black arrow points to a representative spermatogonia, the red arrow, primary spermatocyte, and the green arrowhead, Sertoli cell. In addition, the green curves present regions used to analyze the cross-sectional area of seminiferous tubule whose value displayed beside. **B** The area of seminiferous tubules from different treatment groups. **C** Quantification of spermatogonia, primary spermatocytes and Sertoli cells from different treatment groups. *N* = 6. Bars assigned with different superscripts mean significant difference (*p < 0.05*)

Previous studies have confirmed the causality between DM and male reproductive dysfunction [7]. To identify the protective role of ICA on male reproductive function, we first examined testis weight, epididymis weight, sperm number and sperm motility. Compared with T2DM group, there were obvious recovery in the four former parameters of both Metformin group and ICA group (Table 3), though the increase of the sperm number in ICA group was not so comparable to that of Metformin group (Table 3). Then we further checked the histological structure of seminiferous epithelium. In T2DM group, the seminiferous tubules were severely atrophied to thin tube wall and the cross-sectional area of seminiferous tubule were significantly decreased (Fig. 2A and B). Within the inordinate seminiferous epithelium (Fig. 2A), the number of spermatogonia, primary spermatocytes and Sertoli cells were markedly reduced (Fig. 2C), and there were rare sperm in the lumen (Fig. 2A). In Metformin and ICA groups, however, the histological defects in male diabetic rats were obviously repaired (Fig. 2), especially the number of spermatogonia, primary spermatocytes and Sertoli cells (Fig. 2C)*.*

**Table 3:** Effect of ICA on testis weight, epididymis weight, sperm number and sperm motility

Group	Testis Weight (g)	Epididymis Weight (g)	Sperm number (×10^6^)	Sperm motility (%)
Control	1.735 ± 0.043^a^	0.7274 ± 0.048^a^	154.7 ± 21.85^a^	13.46 ± 3.29^a^
T2DM	1.228 ± 0.230^b^	0.3805 ± 0.0656^b^	31.40 ± 14.20^b^	4.13 ± 0.91^b^
Metformin	1.657 ± 0.079^a^	0.5634 ± 0.0464^a^	230.7 ± 72.6^c^	13.10 ± 1.89^a^
ICA	1.516 ± 0.143^a^	0.4935 ± 0.0328^a^	132.6 ± 71.65^a^	12.27 ± 5.380^a^

In each column, values assigned with different superscripts indicate significant difference (*P* < 0.05)

### ICA upregulated the expression of proliferative PCNA

**Fig. 3:**
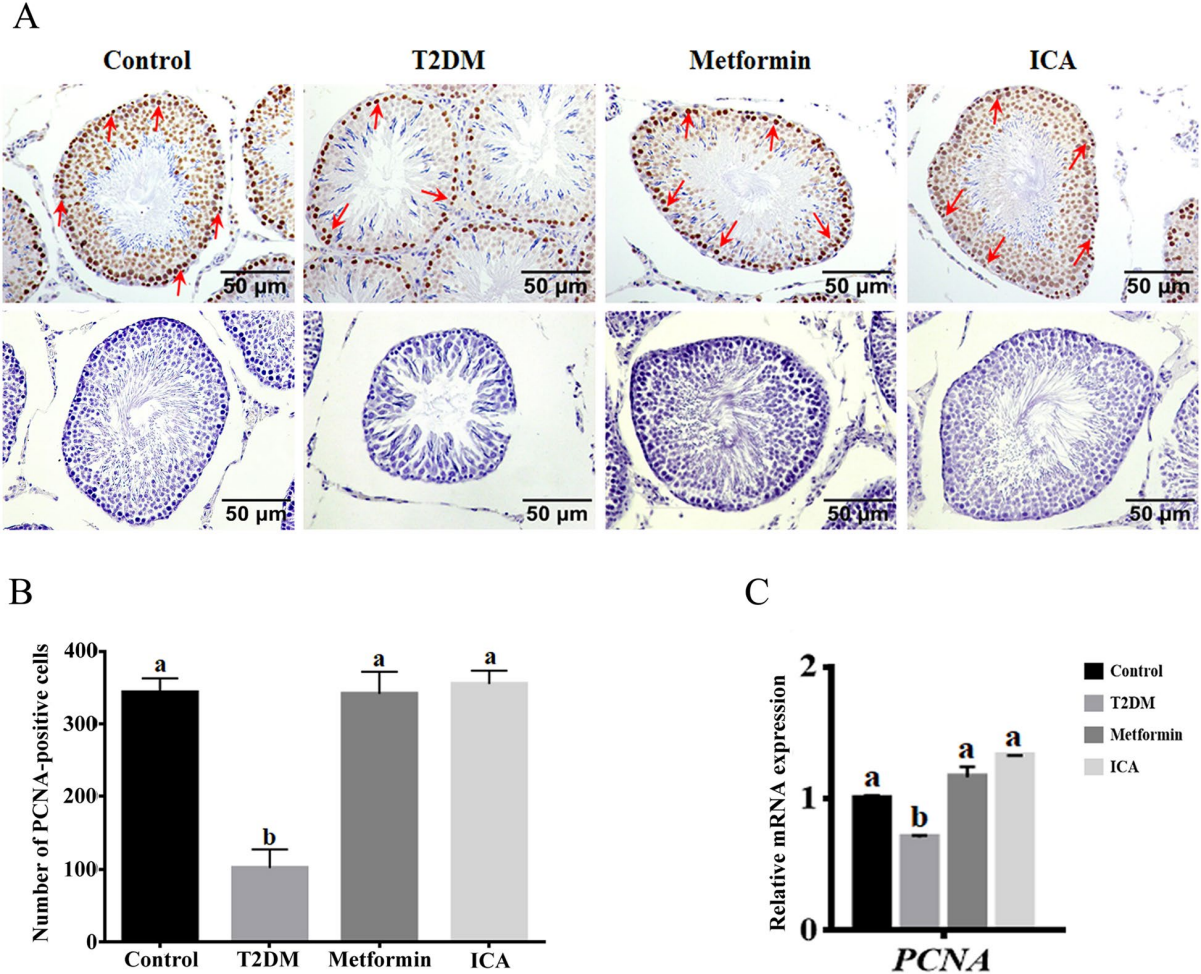
ICA administration upregulated the expression of PCNA in the testis of diabetic rats. **A** Immunohistochemical analysis of PCNA expression. Red arrows point to representatively positive signals of spermatogonia. **B** Quantification of PCNA positive cells from different groups. **C** mRNA expression analysis of *Pcna* with qRT-PCR. The upper panes in A mean PCNA positive cells, and the lower panes present negative controls. *N* = 6, Bars assigned with different superscripts mean significant difference (*p < 0.05*)

**Fig. 4:**
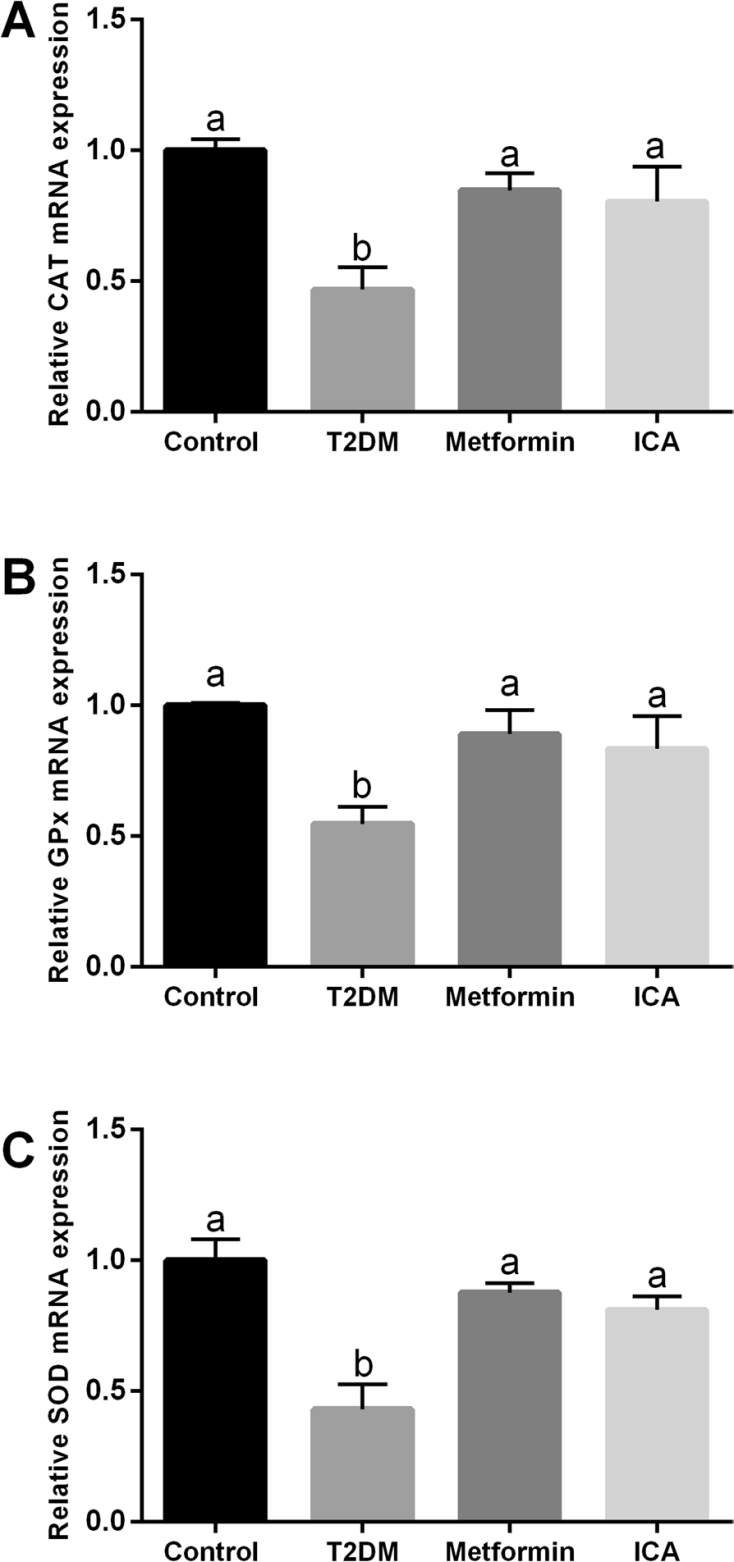
Effects of ICA administration on the mRNA expression levels of CAT, GPx and SOD in the testis of diabetic rats. The mRNA expression levels of (**A**) CAT, (**B**) GPx and (**C**) SOD in the testis are shown. *N* = 6. Bars assigned with different superscripts mean significant difference (*p* < 0.05)

Furthermore, we examined the distribution and expression of Proliferating cell nuclear antigen (PCNA), which plays a crucial role in the balance of cell survival and death [45, 46]. Our results showed PCNA was detected in nearly all spermatogenic cells and Sertoli cells, and the strongest signal of PCNA staining was observed in the perimeter zone of seminiferous tubule where spermatogonia, primary spermatocytes and Sertoli cells mainly reside in (Fig. 3A). In T2DM group, the number of PCNA positive cells (Fig. 3B) and mRNA expression of *Pcna* (Fig. 3C) were significantly reduced and downregulated respectively. However, after the treatment of ICA, the PCNA signal and PCNA-positive cells were observably increased (Fig. 3A and B). Furthermore, the mRNA expression of *Pcna* were also markedly upregulated (Fig. 3C). Expectably, metformin administration showed similar effect when compared with that of ICA (Fig. 3). In addition, our qPCR results revealed that the mRNA expression levels of catalase (CAT), glutathione peroxidase (GPx) and superoxide dismutase (SOD) were significantly down-regulated in the testis of T2DM group when compared to control group (Fig. 4), and metformin and ICA treatment both partially restored the mRNA expression levels of CAT, GPx and SOD in the testis of diabetic rats (Fig. 4).

### ICA activated SIRT1-HIF-1α axis

**Fig. 5:**
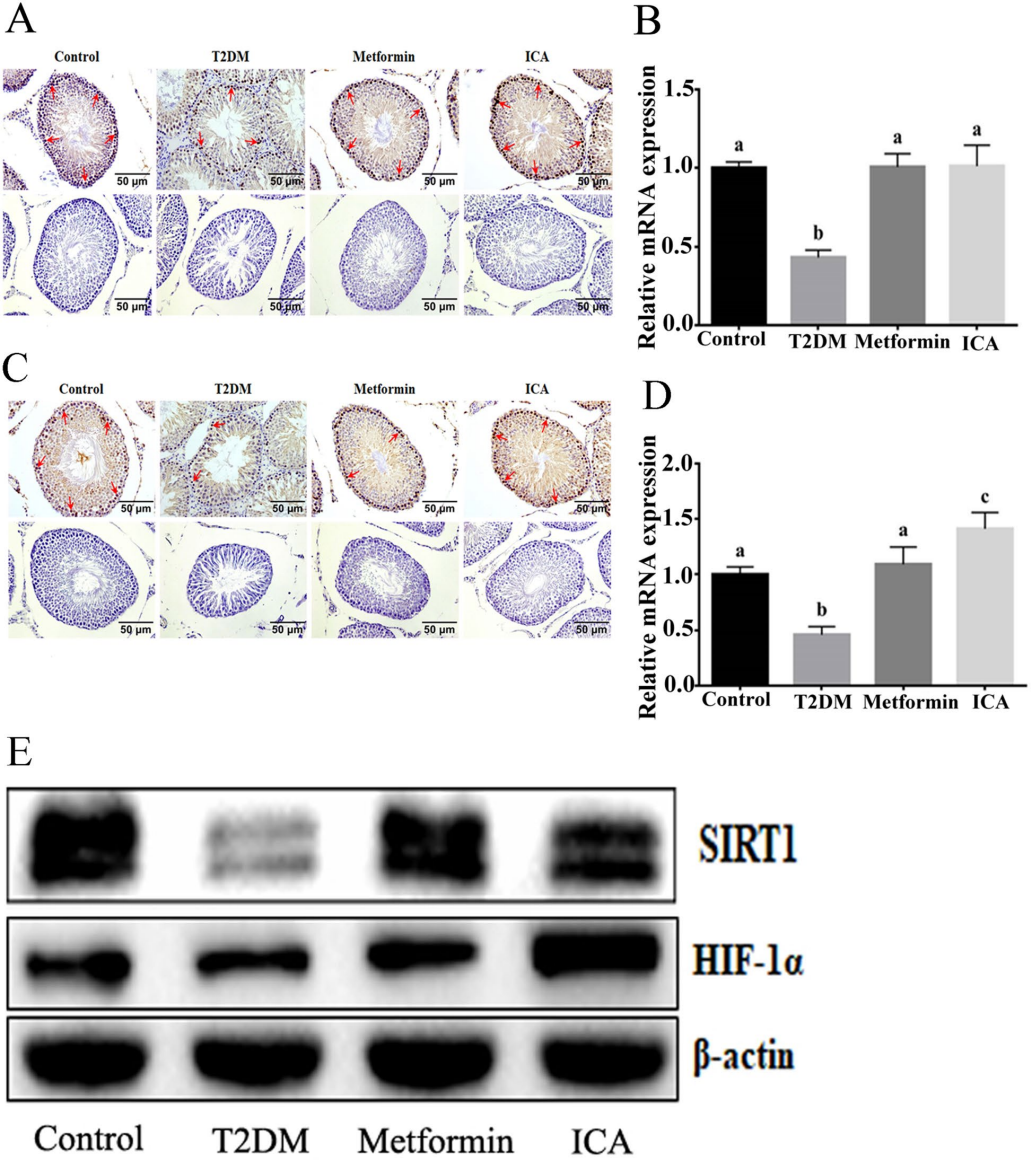
ICA administration upregulated the expression of SIRT1 and HIF-1α in the testis of diabetic rats. **A** Immunohistochemical analysis of SIRT1 expression, and red arrows show representatively positive signals of spermatogonia. **B** mRNA expression analysis of *Sirt1* with qRT-PCR. **D** Immunohistochemical analysis of HIF-1α expression. Red arrows show representatively positive signals of spermatogonia. **D** mRNA expression analysis of *Hif-1α* with qRT-PCR. **E** Western blotting analysis of SIRT1 and HIF-1α protein expression. Bars assigned with different superscripts mean significant difference (*p < 0.05*)

Growing studies indicated that SIRT1, one of the most studied sirtuins, plays vital roles in spermatogenesis and male reproductive functions [47–51]. There is an apparent correlation between SIRT1 and insulin resistance [52]. In addition, SIRT1 could directly deacetylate and stabilize HIF-1α [53, 54], a hypoxia activated transcription factor [55] that regulates the transcriptional response of hypoxia-responsive factors to protect spermatogenic cells against apoptosis in testis [56–58]. These results suggested an important protective roles of SIRT1-HIF-1α axis in repairing DM-induced male reproductive dysfunction [14]. Consequently, we further checked the activity of SIRT1-HIF-1α axis. In T2DM group, the number of SIRT1 positive and HIF-1α positive cells were both reduced (Fig. 5A and C). qRT-PCR and western blotting analysis showed an identical downregulation of *Sirt1* and *Hif-1α* (Fig. 5B, D and E). In contrast, ICA or metformin administration observably reversed these disorders, not only enhanced the signal of SIRT1 and HIF-1α (Fig. 5A and C) but also upregulated the expression of *Sirt1* and *Hif-1α* (Fig. 5B, D and E).

### ICA alleviated mitochondrial apoptosis signaling in diabetic rats

**Fig. 6:**
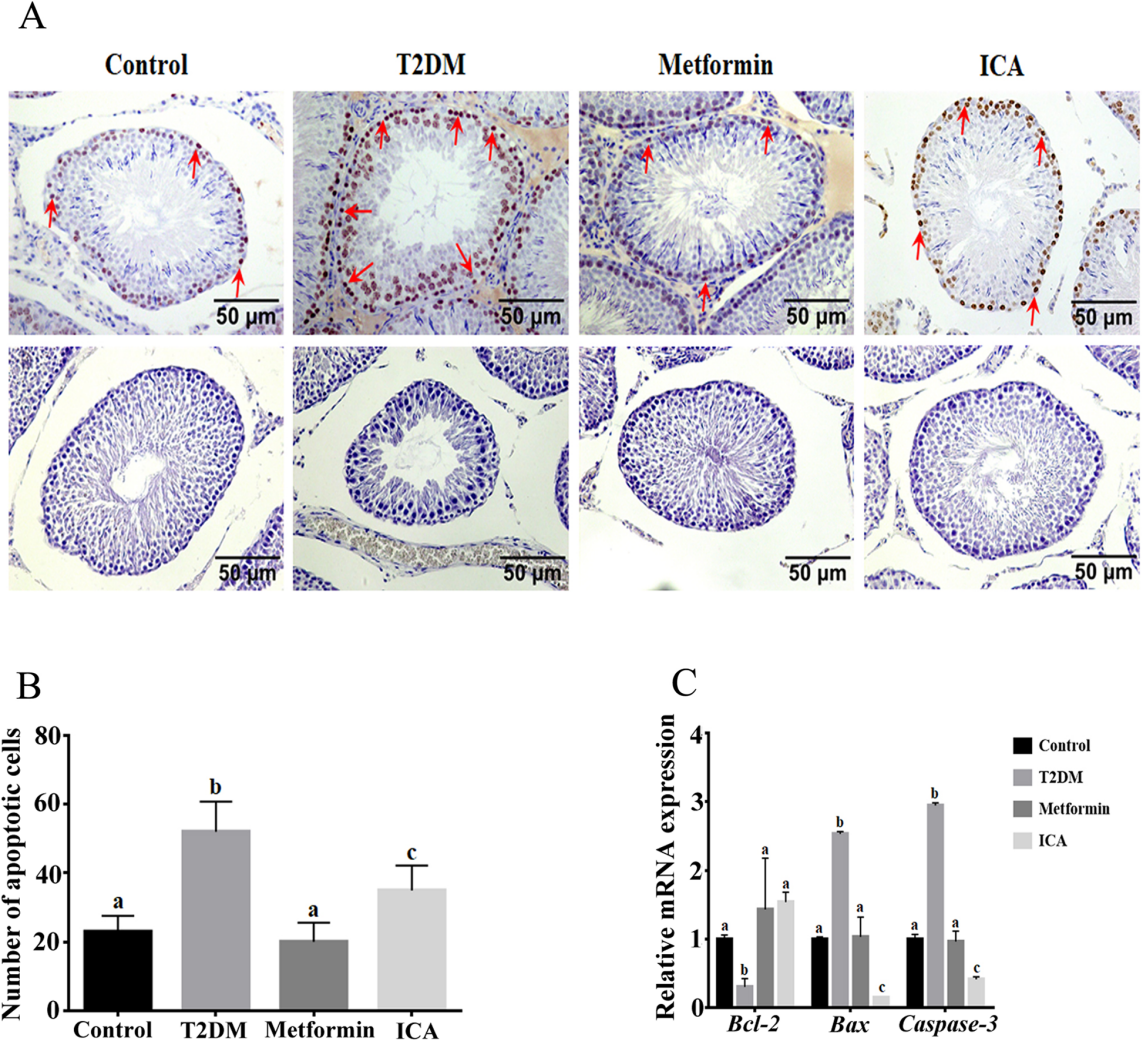
ICA administration alleviated the cell apoptosis in the testis of diabetic rats. **A** TUNEL assay of testicular tissues from different groups. Red arrows show representatively apoptotic signals of spermatogonia. **B** Quantification of apoptotic cells in A. **C** mRNA expression analysis of *Bcl-2, Bax and Caspase 3* with qRT-PCR. The upper panes in A show cells with apoptotic signal, and the lower panes present negative controls. Bars assigned with different superscripts mean significant difference (*p < 0.05*)

Normal development of germ cells and reproductive system highly relies on balanced regulation of mitochondrial apoptosis by BCL-2 family proteins [38]. Several studies have proved the close relationship between the anti-apoptotic effect of ICA and Bcl-2 signaling pathway [15, 35, 59]. Consequently, we also conducted TUNEL assay and identified the expression of mitochondria apoptosis pathway related genes, including *Bcl-2*, *Bax* and *Caspase-*3 (Fig. 6). Compared with the control group, the number of apoptosis cells in the seminiferous tubules of T2DM group were significantly increased, with apoptotic signal mainly focusing on the perimeter area (Fig. 6A and B). Furthermore, the mRNA expression of *Bcl-2* and *Bax* and *Caspase-*3 showed significant downregulation and upregulation respectively (Fig. 6C). In contrast, in ICA and Metformin groups, the number of apoptotic cells were obviously decreased (Fig. 6A and B), and the mRNA expression of *Bcl-2* and *Bax* and *Caspase-*3 were significantly upregulated and downregulated respectively, especially in ICA group (Fig. 6C).

## Discussion

### ICA, DM and male reproductive dysfunction

Owning to complex complications, DM can severely impair reproduction [7, 9]. Hyperglycemia in DM can dramatically cause histological damages to the seminiferous tubular of testis [11, 12] including a reduction in the size of the seminiferous tubules and degeneration of spermatogonia and spermatocytes [60]. Hyperglycemia can also decrease sperm counts, sperm motility and semen volume [61, 62]. In this study, HFD and STZ induced diabetic rats presented severely histological impairment, including reduced cross-sectional area and morphological disruption of seminiferous tubules and decreased number of spermatogonia, primary spermatocytes, Sertoli cells and sperm. In addition, significant increase in FBG and IRI, decrease in testis weight, epididymis weight, sperm number and sperm motility were also found. ICA administration effectively improved these male reproductive damages of diabetic rats.

### ICA and proliferation

PCNA plays a vital role in the proliferation of spermatogonia, primary spermatocytes and Sertoli cells [63]. Indeed, HFD and STZ-induced diabetic rats showed dramatical downregulation of PCNA expression. This downregulation may lead to the dysfunction of combination of replication factors and proteins that involving in DNA replication, cell cycle control and repair, eventually causing the observable decline of spermatogonia, primary spermatocytes and Sertoli cells. ICA or metformin treatment not only recovered the expression of PCNA, but also increased the number of spermatogonia, primary spermatocytes and Sertoli cells.

SIRT1, mainly expressed in spermatocytes and spermatogonia, has been implicated in the germ cell development of males [50, 51]. Sirt1 deficiency was found to attenuate spermatogenesis and germ cell function [64], delay the differentiation of spermatogenic stem cells [51], decrease sperm counts [64] and increase DNA damages/lesions [51] and the apoptosis of pachytene spermatocytes [50]. Here, there were significant decrease in the expression of SIRT1 and reduction in the number of spermatogonia and primary spermatocytes in diabetic rats, which were consistent with previous study. After the treatment with ICA or metformin, the expression of SIRT1 and the number of spermatogonia and primary spermatocytes were observably upregulated and increased respectively, which indicated that ICA may improve DM-induced male reproductive dysfunctions potentially via upregulating the activity of SIRT1. Interestingly, though the treatment of metformin significantly increased FINS, ICA administration showed no significant difference to T2DM group. This may be due to the higher efficiency of ICA in protecting against IRI than metformin. In addition, Dodd et al., found the abnormal expression of HIF-1α, a direct target of SIRT1 [53, 54] in diabetic rats [65]. After the treatment with ICA or metformin, there was a significant upregulation of HIF-1α. Notably, this upregulation in ICA group showed an observable excess to that of Metformin group, indicating a possibly better efficiency of ICA in anti-hypoxia to that of metformin.

### ICA and apoptosis

Apoptosis, a programmed cell death, is crucial for normal development and for maintaining the homeostasis of organism [38]. Proapoptotic protein Bax can activate mitochondria-dependent caspase signaling pathway to cause apoptosis. Anti-apoptotic protein Bcl-2 prevents Bax from translocating to mitochondria to inhibit apoptosis [66–68]. The normal development of germ cells and reproductive system highly relies on the balanced regulation of mitochondrial apoptosis [38]. There were severe impairment of mitochondrion and endoplasmic reticulum in spermatogenic cells and Sertoli cells of diabetic rats [69]. However, there was no significant increase in the apoptosis of germ cell of the STZ-treated mice without hyperglycemia [70]. Interestingly, diabetic mice that were treated with insulin on 3d after STZ treatment displayed reduced elevation of whole-body glucose levels and inhibition of apoptotic cell death in the testis [71]. This suggests a close link between hyperglycemia and germ cell apoptosis in diabetes. Here, obvious apoptosis phenomenon was mainly found in perimeter region (near the basal membrane of seminiferous tubules) where mainly contains spermatogonia, primary spermatocytes and Sertoli cells. Importantly, these diabetic rats, with increased apoptosis in spermatogenic cells and Sertoli cells, presented severe hyperglycemia. In addition, TUNEL assay indicated that ICA or metformin administration significantly decreased the cell apoptosis in testis of diabetic rats. Both ICA and metformin treatments dramatically upregulated the expression of *Bcl-2* and downregulated the expression of *Bax* and *Caspase-3* respectively, which is consistent with previous studies [33, 59]. Notably, the expression of *Bax* and *Caspase-3* in ICA group were obviously lower to that of Metformin group, which may be owning to that ICA not only upregulated the expression of Bcl-2 but also directly targeted BAX and inhibited the translocation of Bax to mitochondria [59]. These results indicated that ICA showed better efficiency in attenuating the abnormal apoptosis of DM-induced male reproductive dysfunction. Nonetheless, comparing with Metformin group, though presented lower expression of *Bax* and *Caspase-3* in the testis of diabetic rats, the ICA group showed apparently more apoptotic cells whose underlying mechanisms needs to be further investigated.

### Likely stimulation of ICA on the steroidogenesis and HPG axis

It is well known that sexual hormone regulation is indispensable to maintain normal reproductive function [72]. Studies including clinical and animal models indicated a decreased serum levels of FSH, LH and testosterone in diabetes or diabetic animals [10, 13]. However, ICA could present testosterone mimetic properties and upregulate the expression of testosterone and gonadal androgen receptor gene [21]. Chen et al., found that ICA not only increased the production of testosterone by regulating the expressions of StAR and PBR, but also improved spermatogenesis via regulating the expression of FSHR and claudin-11 [19]. Consequently, ICA could stimulate the secretion of male hormones and increase genital indexes through regulating the HPG axis [28]. In this study, our results showed that ICA upregulated the serum levels of testosterone, LH and FSH, which may facilitate the recovery of male reproductive functions in diabetic rats.

### Study limitations

The present study lacks the gain- or loss-function studies by using activators or inhibitors of the above investigated signaling pathways, which further revealed the mechanistic actions of ICA. The study examined the mRNA expression levels of CAT, GPx and SOD in the testis, however, due to the limited samples, the levels of ROS production have not been examined our studies. The present study has not performed the detailed analysis in types of cells such as interstitial cells in the testis, which should be further explored in the future studies.

## Conclusion

**Fig. 7:**
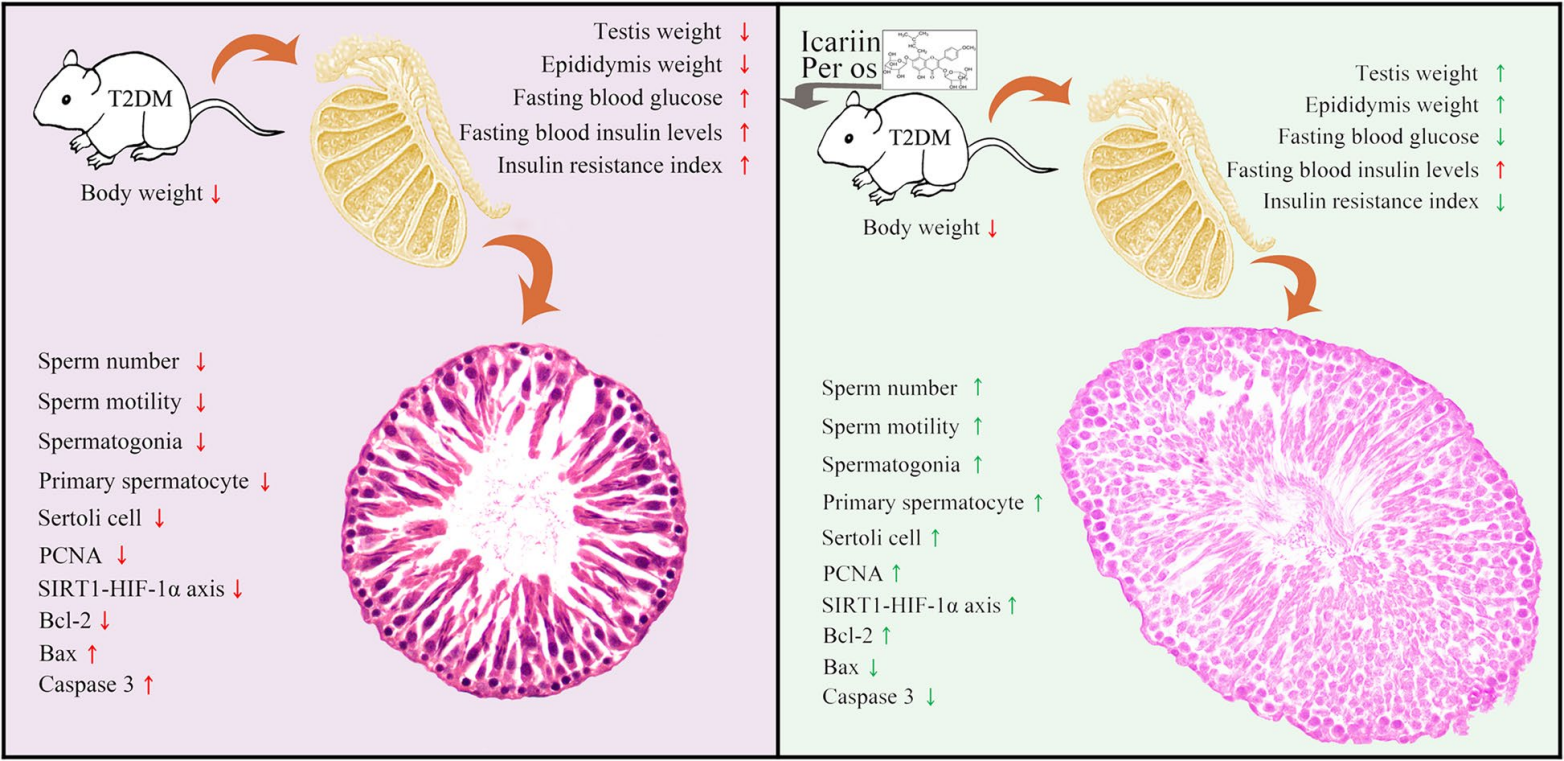
Schematic illustrating ICA’s protection against diabetes mellitus induced testicular dysfunction in rats possibly via enhancing cell proliferation and inhibiting intrinsic mitochondria dependent apoptotic signaling. Red arrows in T2DM panel mean deleterious effects (with significant difference) to reproductive function and relative parameters when compared with normal control group (nondiabetic). Red and green arrows in ICA treatment panel represent that there are no and obvious effectiveness respectively after ICA administration when compared with T2DM group

In conclusion, this study revealed that ICA can protect against DM-induced testicular dysfunction in rats possibly via enhancing cell proliferation and inhibiting intrinsic mitochondria-dependent apoptotic signaling respectively (Fig. 7). Our results demonstrate that ICA effectively attenuated DM-induced male reproductive dysfunctions and spermatogenesis deficiency. ICA may ameliorate DM-induced spermatogenesis deficiency possibly through increasing proliferation and inhibiting intrinsic mitochondria-dependent apoptotic pathway of spermatogonia, primary spermatocytes and Sertoli cells, respectively. Our findings suggested that ICA may be a potentially novel therapeutic agent for the protection and treatment of DM-induced testis damage.

## Data Availability

All the data were available upon the request from the corresponding authors.
